# Extending the Self-Templating Strategy to Micron-Sized Stöber Silica: A Critical Temperature Threshold for Macro/Mesoporous Silicon Anodes

**DOI:** 10.1021/acsomega.6c00506

**Published:** 2026-03-19

**Authors:** Xiuxia Zuo, Shanshan Yin, Suzhe Liang, Ya-Jun Cheng

**Affiliations:** † School of New Energy, 165069 Ningbo University of Technology, Ningbo, Zhejiang 315211, P. R. China; ‡ Ningbo Institute of Materials Technology and Engineering, 74748 Chinese Academy of Sciences, Ningbo, Zhejiang 315201, P. R. China; § The School of Mathematics and Physics, 105810 Jiangsu University of Technology, Changzhou, Jiangsu 213001, P. R. China; ∥ Ningbo Institute of Digital Twin, Eastern Institute of Technology, 639934 Eastern Institute for Advanced Study, Ningbo, Zhejiang 315200, P. R. China; ⊥ College of Renewable Energy, Hohai University, Changzhou, Jiangsu 213200, P. R. China

## Abstract

Silicon anodes are a promising high-capacity alternative to graphite for next-generation lithium-ion batteries, yet their practical application faces challenges due to significant volume changes during cycling. Introducing porous structures can effectively alleviate mechanical stress and enhance cycling stability, which can be realized via magnesiothermic reduction of silica based on a self-templating mechanism. Nevertheless, extending it to solid, monodisperse Stöber silica spheres >500 nm presents a major challenge, wherein elongated magnesium diffusion paths cause incomplete conversion and fragmentation, inhibiting the formation of the well-defined 3D macro/mesoporous networks derived from smaller silica templates. Here, we overcome this limitation by identifying 900 °C as the critical minimum temperature that enables the self-templating mechanism for 730 nm Stöber silica. The enhanced magnesium vapor diffusion and silicon sintering kinetics at this temperature overcome diffusion barriers, generating a well-defined macro/mesoporous network. This optimized porous silicon anode achieves improved cycling stability and rate performance over commercial microsized silicon and lower-temperature samples, maintaining a reversible capacity of 655 mAh g^–1^ after 300 cycles at 0.2C and delivering 656 mAh g^–1^ at 1C. This study reveals temperature as the decisive factor in achieving a diffusion-sintering balance, enabling the successful fabrication of robust, well-defined 3D macro/mesoporous networks from large solid Stöber silica via self-templating.

## Graphical abstract

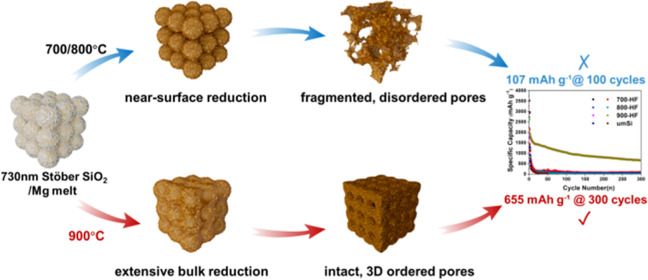

## 1 Introduction

Lithium-ion batteries (LIBs) have become indispensable for portable electronics and electric vehicles (EVs), driven by their unmatched energy density, long cycle life, and environmental benignity. However, the pursuit of next-generation EVs requiring >500 Wh kg^–1^ energy density has exposed critical limitations of conventional graphite anodes (372 mAh g^–1^).^1–3^ Silicon (Si) has emerged as the most promising alternative owing to its exceptionally high theoretical specific capacity (∼3580 mAh g^–1^, ∼10 times higher than graphite), and low lithiation voltage (∼0.4 V vs Li^+^/Li), and natural abundance.^4–6^ Despite these advantages, its commercialization remains hindered by severe volume expansion (∼300–400%) during lithiation/delithiation, which induces particle pulverization, electrical disconnection, and uncontrolled solid electrolyte interphase (SEI) growth, resulting in rapid capacity decay.^7–9^

To mitigate these challenges, extensive efforts have been devoted to strategies such as structural engineering, compositing, binder design, electrolyte optimization and surface/interface modification.^10–15^ Among them, the structural design of silicon itself stands as a fundamental approach, as it directly governs key characteristics such as pore architecture, particle distribution, and electrode integrity, which in turn dictate the electrochemical behavior and the stability of the SEI.^16–18^ Consequently, a variety of silicon architectures have been developed, ranging from nanostructures and core–shell systems to freestanding configurations.^19–22^ In particular, porous Si structures have garnered significant interest due to their unique capability to accommodate large volume changes while maintaining structural stability, thereby effectively mitigating particle pulverization.^23–27^

As a scalable and cost-effective route to synthesize porous silicon, magnesiothermic reduction of silica (SiO_2_) has attracted considerable interest, primarily due to its self-templating capability.^28–31^ This process allows the direct conversion of various silica templates—retaining their morphology—into porous Si frameworks. A wide range of precursors have been explored, including biomass, diatomite, zeolites, and sand.^32–36^ However, the final silicon structure is predominantly determined by the inherent porosity, irregular morphology, and chemical heterogeneity of these precursors, rather than by the reaction conditions. This makes it challenging to decouple the influence of precursor morphology from that of reaction kinetics, consequently impeding the precise and reproducible synthesis of well-defined porous architectures.

In contrast, synthetic Stöber-type SiO_2_, featuring monodisperse spherical morphology and precisely adjustable particle sizes, provides an ideal model system to study the fundamental mechanisms of the self-templating process and to engineer ordered porous silicon with tailored hierarchies.^37–39^ In such hierarchical porous Si, macropores (>50 nm) primarily serve to accommodate the large volume expansion of silicon, while mesopores (2–50 nm) facilitate electrolyte infiltration and enhance ion-transport kinetics—contributing synergistically to structural and electrochemical stability. Our previous studies have demonstrated that Stöber silica particles with diameters of 130 and 420 nm can serve as effective self-templating precursors via magnesiothermic reduction, yielding three-dimensional (3D) interconnected macro-/mesoporous Si with superior cycling stability compared to conventional nano- or micron-sized Si.^40–42^ However, extending this strategy to larger submicron precursors (>700 nm) remains challenging. While the use of large silica sources like diatomite is documented, these are typically porous or irregular, facilitating reactant penetration. In contrast, for large, solid, monodisperse spheres, the increased solid-state diffusion distances for Mg vapor and reaction intermediates within the particle volume are anticipated to weaken the self-templating effect, potentially leading to incomplete conversion and structural collapse during reduction.

This solid-state diffusion limitation is directly supported by kinetic studies of the magnesiothermic reaction, which have established the process as mass-transfer-limited, with the precursor size being a critical kinetic variable that significantly influences the reaction rate.^30,43,44^ This presents a clear and unresolved research question for a specific class of precursors: for large, solid, monodisperse Stöber silica spheres that present no internal porosity to shorten diffusion distances, does a critical reduction temperature threshold exist that provides sufficient kinetic driving force to overcome this intrinsic limitation and enable successful self-templating?

To bridge this gap, we systematically investigated the magnesiothermic reduction of 730 nm Stöber SiO_2_. By correlating the reduction temperature (700 °C, 800 °C, 900 °C) with the structural evolution and electrochemical performance, we demonstrate that a critical minimum temperature of 900 °C is essential for achieving a robust 3D macro-/mesoporous architecture. At this temperature, the drastically enhanced kinetics of Mg vapor penetration and Si domain sintering enable the formation of such a structure. This work breaks the previously known size limit for solid spherical precursors in the self-templating strategy, establishing a clear temperature-size-structure relationship that offers a generalizable kinetic principle for the design of hierarchical porous materials.

## 2 Experimental Section

### 2.1 Preparation of 730 nm Stöber SiO_2_

The 730 nm monodisperse SiO_2_ spheres were produced following a procedure adapted from the classical Stöber synthesis.^45–47^ The synthesis began with the preparation of a homogeneous solution comprising ethanol (50 mL) and ammonium hydroxide (9.5 mL, 25–28%, Sinopharm). This mixture was agitated at 400 rpm, followed by the slow addition of tetraethyl orthosilicate (TEOS, 5 mL, ≥98%, Sinopharm) dissolved in ethanol (30 mL) using a syringe pump. After being stirred continuously for 15 h at room temperature, the SiO_2_ microspheres were collected by centrifugation, followed by ethanol washing and vacuum drying at 80 °C.

### 2.2 Preparation of Porous Silicon

Porous silicon was prepared via magnesiothermic reduction of the as-prepared Stöber SiO_2_, adapting a reported method.^39^ Specifically, a mixture of SiO_2_ and Mg powders with a 1:1 weight ratio was placed in an crucible boat and heated in a tube furnace to the target temperature (700, 800, or 900 °C) at a ramp rate of 5 °C min^–1^, then maintained for 16 h under an Ar/H_2_ (95:5, v/v) flow. The product was then treated with 1 M HCl for 6 h to dissolve MgO and residual Mg, followed by washing and overnight drying at 80 °C to obtain the intermediate samples (700-HCl, 800-HCl, 900-HCl). The intermediates were further etched in 4 wt % HF for 1 h to remove unreacted SiO_2_, yielding the final porous silicon samples (700-HF, 800-HF, 900-HF).

### 2.3 Material Characterization

The crystallographic structure of the samples was analyzed by X-ray diffraction (XRD, Bruker D8 Advance) with Cu Kα radiation. Morphology and microstructure were examined using scanning electron microscopy (SEM, Hitachi S-4800) and transmission electron microscopy (TEM, JEOL JEM-2100F). Specific surface area and pore size distribution were determined by N_2_ adsorption–desorption measurements (Micromeritics ASAP 2020) using the Brunauer–Emmett–Teller (BET) and Barrett–Joyner–Halenda (BJH) methods.

### 2.4 Electrochemical Tests

The electrochemical performance was evaluated using CR2032 coin cells with lithium metal as the counter/reference electrode. The working electrode was prepared by coating a slurry of active material (porous Si), conductive carbon black (Super P, Alfa Aesar), and sodium alginate binder (Aladdin) in a weight ratio of 7:1:1 onto copper foil, followed by drying at 80 °C under vacuum. The typical mass loading density regarding the silicon content was controlled to be around 1.0 mg cm^–2^. The electrolyte was 1 M LiPF_6_ in a mixture of ethylene carbonate and dimethyl carbonate (EC/DMC, 1:1 v/v) with 5 wt % fluoroethylene carbonate (FEC) as an additive. Galvanostatic charge/discharge tests were conducted on a LAND CT2000A system between 0.005 and 3.0 V at 0.2C (1C = 1000 mA g^–1^). Rate performance was assessed at current densities ranging from 0.1 to 1C. Cyclic voltammetry (CV) was performed using a Solartron 1470E system at a scan rate of 0.2 mV s^–1^ between 0.001 and 3.0 V vs Li^+^/Li for the first three cycles.

## 3 Results and Discussion

The structural evolution from SiO_2_ to crystalline Si is clearly demonstrated by the X-ray diffraction (XRD) patterns in Figure 1. As exemplified by the reduction process at 900 °C (Figure 1a), the precursor SiO_2_ exhibits a broad hump centered around 2θ = 20–27°, confirming its amorphous nature. Following magnesiothermic reduction, the as-reduced coarse product displays distinct diffraction peaks assignable to MgO (2θ = 36.8°, 42.9°, 62.3°, 74.7°, and 78.6°), Mg_2_Si (2θ = 24.1°, 39.9°, 57.9°, and 72.1°), and crystalline Si (2θ = 28.4°, 47.3°, 56.1°, 69.1°, 76.4°, and 86.0°), indicating the complete reaction of SiO_2_ with Mg to yield MgO and Si.^40^ Subsequent HCl washing effectively removed the byproduct MgO and Mg_2_Si, as evidenced by the XRD pattern of the resulting sample (900-HCl), which reveals only the characteristic reflections of Si corresponding to the (111), (220), (311), (400), (331), and (422) planes.^40,48^ A final HF wash was applied to eliminate any residual unreacted SiO_2_. The XRD pattern of the purified sample (900-HF) shows solely the typical diffraction peaks of Si. Similarly, as shown in Figure 1b, samples reduced at lower temperatures (700-HF and 800-HF) and subjected to the same purification sequence also exhibit the characteristic Si pattern, with no detectable diffraction signals from MgO or Mg_2_Si byproducts.

**Figure 1:**
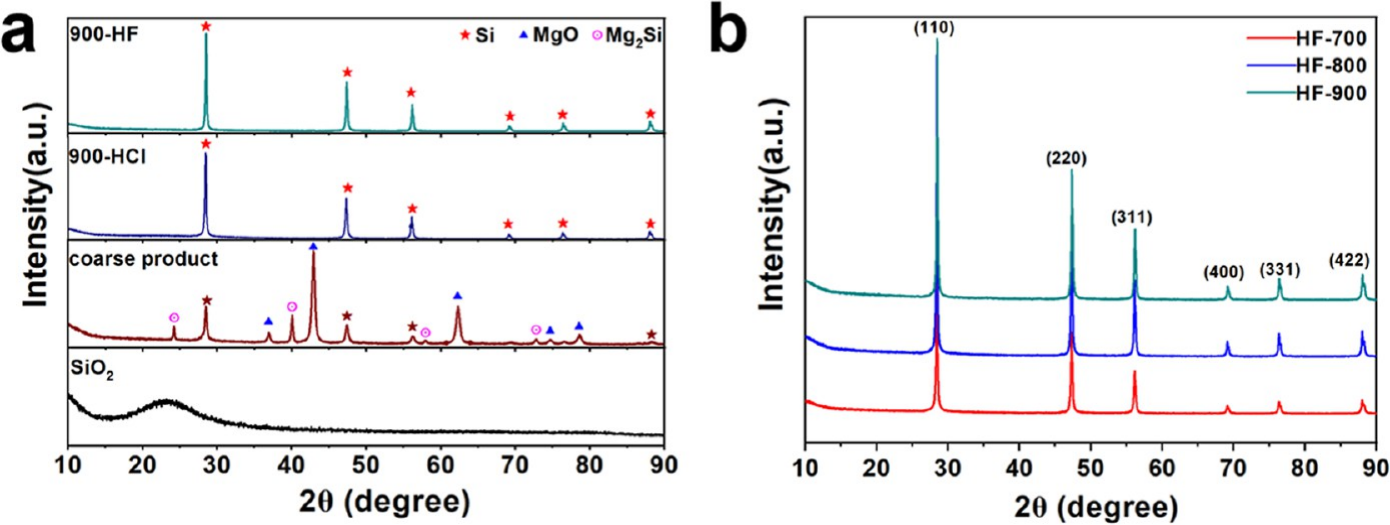
XRD patterns of (a) the Stöber-synthesized SiO_2_ precursor, the as-reduced coarse product, and the corresponding samples after HCl and HF washing (900-HCl and 900-HF); (b) the porous silicon products obtained after reduction at 700, 800, and 900 °C followed by HCl and HF washing (700-HF, 800-HF, and 900-HF).

To investigate the temperature-dependent mechanistic pathways governing morphological evolution, we conducted a systematic microstructural analysis of HCl-washed intermediate products (700-HCl, 800-HCl, 900-HCl) and HF-etched final products (700-HF, 800-HF, 900-HF). Figure 2 provides a comprehensive overview of the stepwise morphological and structural transformations, illustrating how the precursor SiO_2_ evolves through magnesiothermic reduction at 700 °C, 800 °C, and 900 °C, followed by HCl washing to remove reaction byproducts. As depicted in Figure 2a_1_,_2_,b, the pristine Stöber SiO_2_ particles exhibit highly monodisperse spherical morphology with an average diameter of approximately 726 nm. The amorphous nature of the SiO_2_ is confirmed by the diffuse ring in the corresponding SAED pattern (Figure 2c).

**Figure 2:**
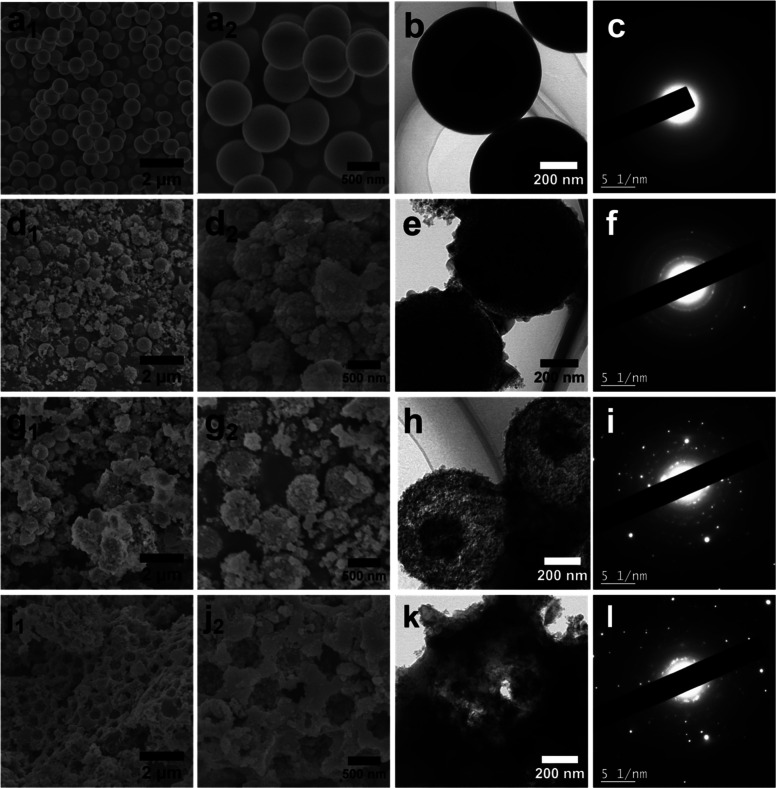
SEM (a_1_, a_2_, d_1_, d_2_, g_1_, g_2_ and j_1_, j_2_), TEM (b,e,h,k) images, SAED patterns (c,f,i,l) of the as-prepared Stöber SiO_2_ (a_1_, a_2_, b, and c), 700-HCl (d_1_, d_2_, e,f), 800-HCl (g_1_, g_2_, h), and (i) 900-HCl (j_1_, j_2_, k,l).

The magnesiothermic reduction temperature profoundly influences the morphology and structural integrity of the resulting silicon products. At 700 °C, the resultant 700-HCl preserves the original spherical morphology of the SiO_2_ precursor and exhibits a rough surface, as evidenced in Figure 2d–e. SEM imaging (Figure 2d_1_,d_2_) also reveals a large number of fragmented particles surrounding the spheres. TEM characterization (Figure 2e) provides further information about the structural transformation—whereas the spherical particles maintain an average diameter of ∼730 nm, closely corresponding to the original SiO_2_ template dimensions, yet display well-developed internal porosity. These voids arise from magnesium-mediated etching of the silica framework, a process that induces both surface roughening and internal void formation. The SAED pattern (Figure 2f) reveals distinct diffraction rings that correspond to the (111) planes of crystalline silicon, indicating the formation of Si nanocrystals even at the relatively low reduction temperature.^40^

Increasing the reduction temperature to 800 °C induces a marked change in morphology. While a minority of intact porous spheres persist, more fractured spheres appeared in the 800-HCl product (Figure 2g_1_,g_2_). TEM imaging (Figure 2h) confirms the porous nature within the spheres. The SAED pattern (Figure 2i) continues to show sharp crystalline rings, signifying well-developed crystallinity.^49^ These observations collectively indicate that reduction at both 700 and 800 °C produces a mixture of intact porous spheres and fragmented particles, with the proportion of fragmentation significantly increasing as the temperature rises from 700 to 800 °C.

A critical morphological transition occurs upon further increasing the reduction temperature to 900 °C. As shown in Figure 2j_1_,j_2_,k, the 900-HCl sample undergoes a fundamental structural reorganization, evolving from discrete spherical particles into a distinctive 3D continuous macroporous network. This interconnected architecture features well-defined macropores with sizes ranging between 400 and 530 nm (Figure 2j_1_,j_2_). TEM image (Figure 2k) provides further evidence of this continuous macroporous framework. The SAED pattern in Figure 2l confirms that the sample remains highly crystalline silicon. This transformation demonstrates that the self-templating mechanism effectively operates at 900 °C, even in the absence of HF etching, driving the formation of a fully interconnected macroporous silicon architecture with high crystallinity.

Figure 3 further presents the morphological and crystalline characteristics of the HF-etched samples (700-HF, 800-HF, and 900-HF). Based on the mass loss after HF etching (approximately 74%, 65%, and 36% at 700 °C, 800 °C, and 900 °C, respectively), the SiO_2_-to-Si conversion yields were calculated to be 42%, 53%, and 79%. The yield represents the percentage of reacted SiO_2_ in the initial mass, derived by assuming the mass loss to unreacted SiO_2_ removed by HF, and converting the remaining Si mass back to reacted SiO_2_ equivalent using the stoichiometric ratio (60/28).^42^ These yields show a clear increasing trend with the reduction temperature, which is closely linked to the distinct morphological evolution observed across the samples.

**Figure 3:**
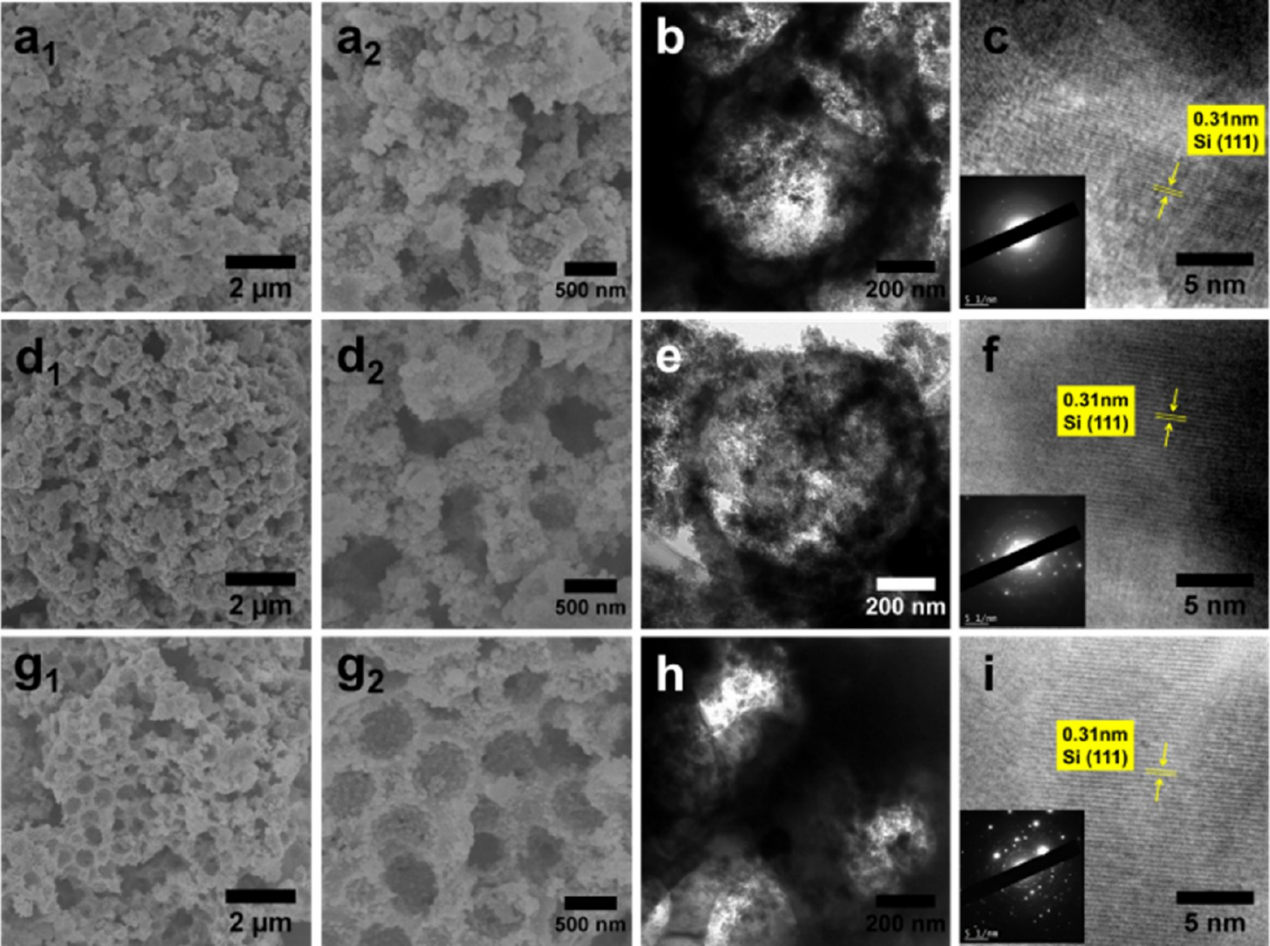
SEM images (a_1_, a_2_ for 700-HF; d_1_, d_2_ for 800-HF; and g_1_, g_2_ 900-HF), TEM images (b for 700-HF; e for 800-HF; h for 900-HF), and corresponding HRTEM images and SAED patterns (c for 700-HF; f for 800-HF; i for 900-HF).

SEM imaging in Figure 3a_1_,a_2_ demonstrate that HF etching converts the 700-HCl intermediate into a porous, granular structure. The particles, spherical or irregular with rough surfaces, interconnect to form a heterogeneous and disordered 3D network, exhibiting a wide range of particle sizes and void spaces. TEM analysis in Figure 3b reveals discrete macropores with diameters around 690 nm, whose formation is attributed to selective dissolution of unreacted SiO_2_ residues within the in situ formed porous silicon network during the reduction process. HRTEM (Figure 3c) shows lattice fringes spaced at 0.31 nm, corresponding to cubic Si (111) planes, while SAED patterns (inset, Figure 3c) display diffraction rings indexed to the same planes.^50^ These results demonstrate that HF treatment effectively exposes the macropores hidden in the 700-HCl intermediate, which contains both a silicon framework and unreduced silica components.

Similar to 700-HF, the SEM images in Figure 3d_1_,d_2_ reveal that 800-HF exhibits a porous, clustered structure with localized macropore clusters, showing significant variation in pore sizes rather than a uniform distribution. TEM imaging in Figure 3e confirms the existence of macropores (∼710 nm) within these domains. HRTEM and SAED (Figure 3f) validate the preserved crystallinity of the silicon framework after etching.

Unlike the 700-HF and 800-HF samples, the 900-HF sample preserves the 3D interconnected macroporous architecture of its 900-HCl precursor (as shown in Figure 3g_1_,g_2_). It presents a highly porous, interconnected framework featuring a continuous open-cell structure with multiscale pore sizes. TEM analysis (Figure 3h) corroborates the interconnected nature of the framework, revealing macropores with diameters ranging from 540 to 600 nm. The reduction in pore size compared to the 800-HF sample is attributed to an enhanced SiO_2_-to-Si conversion efficiency during the reduction process, which results in thicker silicon formations. HRTEM and SAED (Figure 3i) further confirm the intact crystallinity of the silicon matrix, which was established during the high-temperature reduction.

The collective analysis of the HF-etched samples reveals that only reduction at 900 °C produces a robust, pervasive 3D continuous macroporous structure in the final silicon (900-HF), whereas lower temperatures (700 °C, 800 °C) yield only discrete, localized macropores. This demonstrates that the self-templating mechanism, essential for replicating a well-defined, continuous porous architecture from the precursor, operates exclusively at 900 °C for large SiO_2_ precursors (730 nm). Despite successful templating at 900 °C, the resulting macroporous structure exhibits reduced integrity and pore regularity compared to systems using smaller precursors (420 or 130 nm), attributed to greater challenges in achieving the complete and uniform fusion/sintering of in situ formed silicon domains during high-temperature reduction, which results in thicker and less uniform pore walls and diminished long-range order.

To investigate the temperature-dependent evolution of pore architecture during magnesiothermic reduction, nitrogen adsorption–desorption analysis was conducted on all HCl- and HF-treated samples. As illustrated in Figure 4a,b, all isotherms exhibit Type-IV curves with H3-type hysteresis loops at high relative pressures (*P*/*P*_0_ > 0.4), indicative of the existence of mesopores.^51^ The Barrett–Joyner–Halenda (BJH) pore size distributions derived from the adsorption branches (Figure 4c,d) further confirm the dominance of mesopores, with primary peaks located below 50 nm for all samples. Calculated average pore diameters (4V/A by BET surface area) range from 7.8 to 9.2 nm (Table 1), confirming the mesoporous nature. Combined with SEM and TEM observations (Figures 3 and 4), these results demonstrate that 700-HCl and 800-HCl samples exhibit a mesoporous architecture with ∼9 nm pores while 900-HCl, 700-HF, 800-HF, and 900-HF samples develop hierarchical porosity, integrating BJH-confirmed mesopores with SEM-observed interconnected macropores.

**Figure 4:**
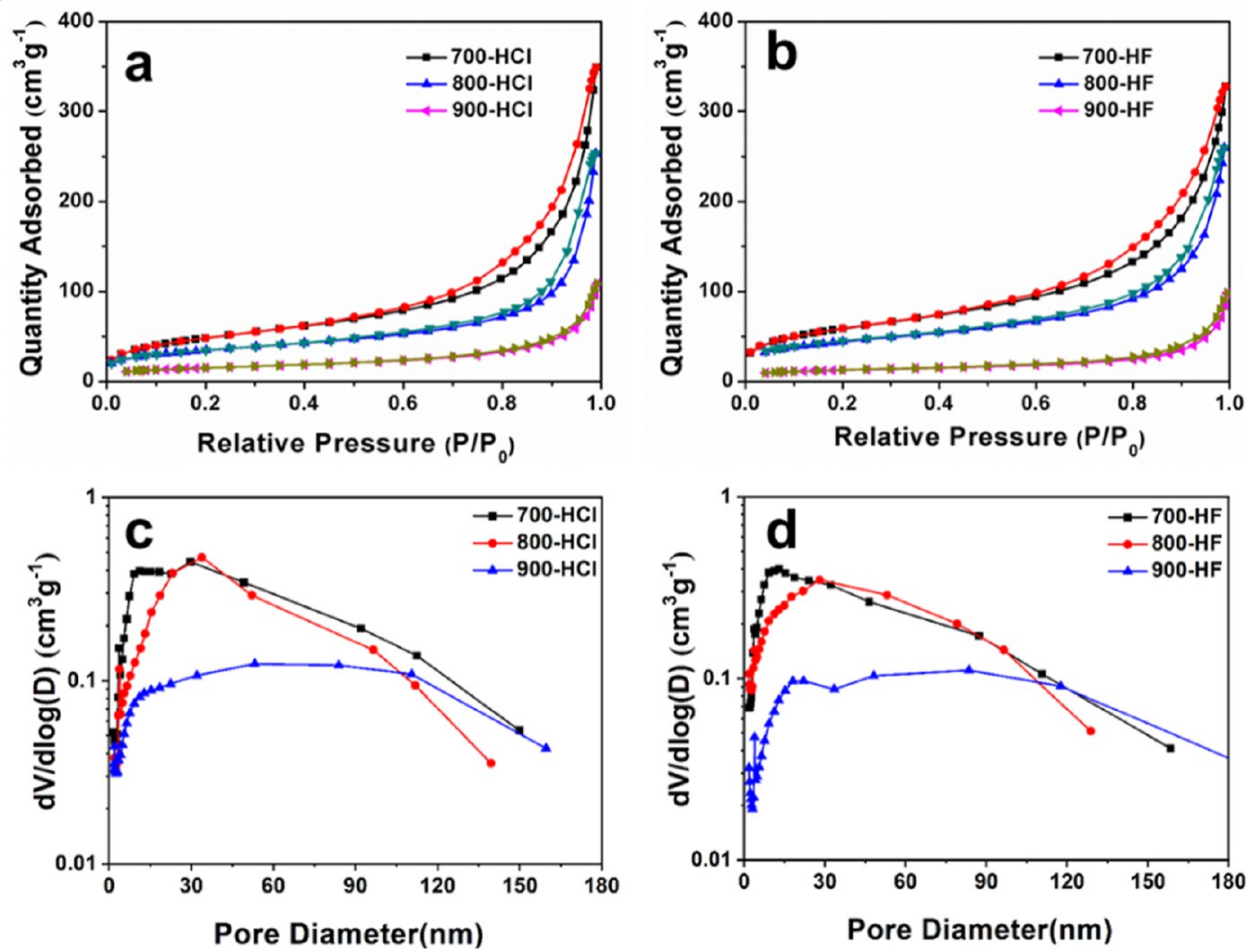
N_2_ adsorption and desorption isotherms (a,b) and the corresponding BJH pore size distribution curves (c,d) of the HCl washed silicon (700-HCl, 800-HCl and 900-HCl) and the HF etched samples (700-HF, 800-HF and 900-HF).

**Table 1:** Porosity Information of Porous Silicon Synthesized with Different Magnesiothermic Reduction Temperatures (700 °C, 800 °C, and 900 °C), Followed by HCl Washing and HF Etching

samples	BET surface area/m^2^ g^–1^	pore volume/cm^3^ g^–1^	average pore diameter/nm	pore distribution/m^2^ g^–1^	
				mesopores (2–50 nm)	macropores (>50 nm)
700-HCl	178.0	0.49	9.1	139.7	6.2
700-HF	212.7	0.53	7.8	160.0	3.7
800-HCl	124.8	0.38	9.2	80.0	4.7
800-HF	158.4	0.39	8.1	126.6	3.2
900-HCl	55.3	0.17	8.1	45.3	2.1
900-HF	45.9	0.15	8.4	32.1	1.6

Detailed analysis shows that the HCl-washed intermediates (700-HCl, 800-HCl, 900-HCl) demonstrate a temperature-dependent trend in their textural properties, where both specific surface area (SSA) and total pore volume (PV) decrease with increasing temperature. At 700 °C, magnesium-induced etching of silica creates numerous mesopores within the particles, including some closed pores (as seen in the TEM images in Figure 2e). Despite poor pore channel connectivity, as indicated by a weak sub-10 nm peak in the BJH curves (implying pore blockage), the high mass fraction of porous particles allows 700-HCl to achieve the highest SSA (178.0 m^2^ g^–1^) and PV (0.49 cm^3^ g^–1^). When the temperature is increased to 800 °C, the improved reduction kinetics significantly enhance the conversion of SiO_2_ to Si, reducing the proportion of residual SiO_2._ This leads to two concurrent structural effects: (i) the closure or coarsening of a significant fraction of high-surface-area mesopores; and (ii) stress-induced macroscopic particle fragmentation resulting from enhanced Si-domain sintering and densification. The fragmented particles contribute minimally to the total BET surface area and cannot compensate for the substantial loss caused by mesopore elimination. These combined effects lead to a noticeable drop in SSA (124.8 m^2^ g^–1^) and PV (0.38 cm^3^ g^–1^) for 800-HCl. Further temperature rise to 900 °C intensifies the reduction process, driving further SiO_2_ conversion, accelerating Si fusion, and depleting residual mesoporous SiO_2_, while also prompting pore structure reconstruction. Consequently, 900-HCl shows a decline in SSA (55.3 m^2^ g^–1^) and PV (0.17 cm^3^ g^–1^), resulting in a densified, low-porosity structure, though the formation of macropores partially balances the overall porosity reduction. This temperature-driven evolution of textural properties in HCl-washed intermediates is in line with that observed in silicon formed from a 420 nm SiO_2_ precursor.

The HF-etched final products (700-HF, 800-HF, 900-HF) demonstrate a parallel decreasing trend in porosity characteristics: SSA declines progressively from 212.7 m^2^ g^–1^ (700-HF) to 158.4 m^2^ g^–1^ (800-HF) and ultimately to 45.9 m^2^ g^–1^ (900-HF), while PV follows a corresponding reduction from 0.53 cm^3^ g^–1^ to 0.39 cm^3^ g^–1^ and 0.15 cm^3^ g^–1^. Since the residual SiO_2_ is completely removed, this systematic reduction primarily arises from the enhanced fusion of the in situ formed silicon. Crucially, HF etching induces a divergent structural response that depends directly on the extent of prior silica-to-silicon conversion. For the 700 and 800 °C systems, the BJH curves show the primary peak shifts slightly toward smaller diameters and the pore-volume contribution in the 2–30 nm range increases after HF treatment. This indicates that HF treatment not only removes unreacted silica but, more importantly, opens previously inaccessible or closed mesopores, which in turn enhances pore interconnectivity, increases accessible surface area, and augments overall pore volume. In stark contrast, for the 900 °C system, the pore size distributions of 900-HCl and 900-HF are nearly identical, with only a slight decrease in intensity after etching. This reflects the near-complete silica-to-silicon conversion prior to etching, leaving minimal SiO_2_ residue to be removed. Consequently, at 900 °C, the dominant effect of HF treatment is limited to the removal of a small mass of residual silica, which cannot compensate for the concurrent sintering-driven densification of the silicon matrix—resulting in an overall decrease in porosity. This clear contrast underscores how the reduction temperature governs the residual SiO_2_ architecture and thereby dictates whether HF etching primarily opens pores (at lower temperatures) or merely removes trace residue while densification proceeds (at 900 °C).

Based on the systematic characterization results, the temperature-dependent structure evolution of porous silicon reduced from 730 nm Stöber SiO_2_, as schematically illustrated in Figure 5, can be rationalized by the interplay between two thermally activated processes: the vapor-phase penetration and reduction by Mg and the solid-state sintering of the newly formed Si. This interplay is critically governed by the precursor size, which imposes a fundamental diffusion constraint.

**Figure 5:**
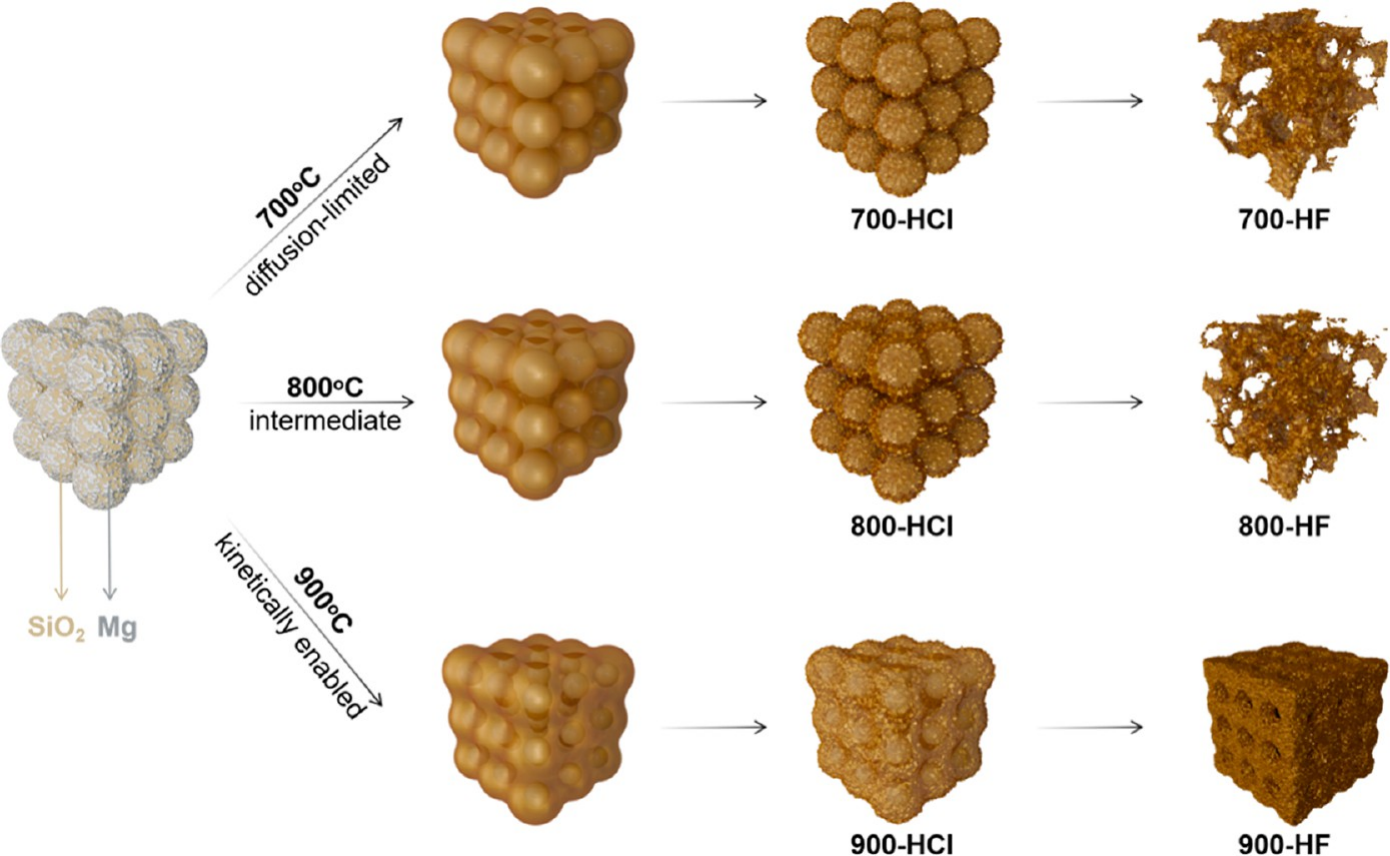
Schematic illustration of the temperature-dependent structural evolution during the magnesiothermic reduction of 730 nm solid Stöber SiO_2_. The process transitions through three distinct regimes: (a) 700 °C (Diffusion-Limited Regime), where surface reaction yields fragmented Si particles and incomplete macroporosity; (b) 800 °C (Intermediate Regime), characterized by deeper yet incomplete reduction and partial sintering leading to fractured particles; and (c) 900 °C (Kinetically Enabled Regime), where sufficient Mg penetration and Si sintering produce a robust, continuous 3D macroporous network.

The magnesiothermic reduction is a well-established diffusion-controlled process.^44,52^ For our system, the key challenge arises from the use of large (730 nm), solid, and monodisperse SiO_2_ spheres, which present elongated diffusion pathways for Mg vapor compared to smaller or intrinsically porous precursors. This size effect creates a substantial kinetic barrier that must be overcome to achieve complete conversion and structural replication.

At 700 °C, the provided thermal energy is insufficient to overcome this high kinetic barrier imposed by the 730 nm solid SiO_2_ spheres. Consequently, Mg vapor penetration is largely restricted to the near-surface region, yielding a fragmented and poorly connected porous silicon framework that encapsulates unreacted silica. Concurrently, the low atomic mobility of silicon under this temperature, coupled with the large curvature of the initially formed Si domains, strongly inhibits their sintering and coalescence among different silica spheres to form 3D connected structures. The coarse reduction product therefore consists predominantly of fragmented silicon particles mixed with partially reacted silica cores. Subsequent HF etching removes the residual silica, exposing a macroporous architecture combined with fragmented particles. This outcome is characteristic of a diffusion-limited, incomplete reaction regime.

At 800 °C, enhanced Mg vapor pressure and Si diffusion allow the reduction front to propagate deeper into the SiO_2_ particles, and sintering becomes more localized. However, these kinetics remain below the critical threshold required for the complete conversion and structural integration of such large, solid precursors. The outcome is still a predominance of fractured particles, demonstrating that the self-templating mechanism fails at this temperature. The persistent fragmentation underscores that the intrinsic diffusion limitation dictated by the precursor size is not yet resolved.

A fundamental transition occurs at 900 °C. This temperature represents the critical minimum threshold at which the provided thermal energy decisively exceeds the activation barrier specific to the 730 nm solid-sphere system. The Mg vapor pressure rises exponentially, enabling rapid and complete penetration through the entire particle volume.^29^ Simultaneously, the sintering kinetics of Si are dramatically enhanced, allowing the in situ-generated Si domains to overcome curvature-induced energy barriers. Widespread grain-boundary migration and coalescence occur, transforming the discrete precursor morphology into a robust, well-defined, and continuous 3D macroporous network that faithfully replicates the spherical template packing. The near-total consumption of SiO_2_ minimizes residual content, rendering the porous Si framework stable against HF etching. This successful structural replication at 900 °C demonstrates that the self-templating mechanism, previously effective mainly for sub-500 nm SiO_2_, can be extended to larger solid precursors provided the temperature is high enough to drive both complete Mg vapor penetration and sufficient Si sintering, thereby overcoming the intrinsic diffusion barrier associated with the precursor’s size and non-porous morphology.

Building on the established temperature-dependent morphological evolution of porous silicon, we systematically evaluated the electrochemical performance of 700-HF, 800-HF, and 900-HF samples as LIB anode materials. Figure 6 presents the comparative electrochemical characterization of these electrodes, with CV profiles (Figure 6a–c) revealing distinct redox behaviors characteristic of silicon-based anodes during initial three cycles. A distinctly broad cathodic peak emerges at approximately 1.2 V only in the first scan, corresponding to electrolyte decomposition and SEI formation, accounting for the initial irreversible capacity loss. A sharp reduction peak appears below 0.1 V in all cathodic scans, corresponding to the well-established conversion of crystalline silicon into amorphous Li_*x*_Si alloys. In the anodic scans, two oxidation peaks emerge at ∼0.34 V and ∼0.54 V, associated with the stepwise delithiation of Li–Si alloys (Li_*x*_Si → Si).^40,53^ Moreover, the current peak intensity is progressive enhanced with cycling, indicating gradual activation of silicon active sites.^48^ This activation is primarily attributed to the progressive breakdown of cubic silicon structure, which depends significantly on the diffusion rate of Li^+^ into porous silicon particles and the formation rate of amorphous Li–Si phases.^18^ Overall, the electrochemical reaction mechanism of porous silicon derived at 700–900 °C closely resembles that of commercial microsized silicon anodes.

**Figure 6:**
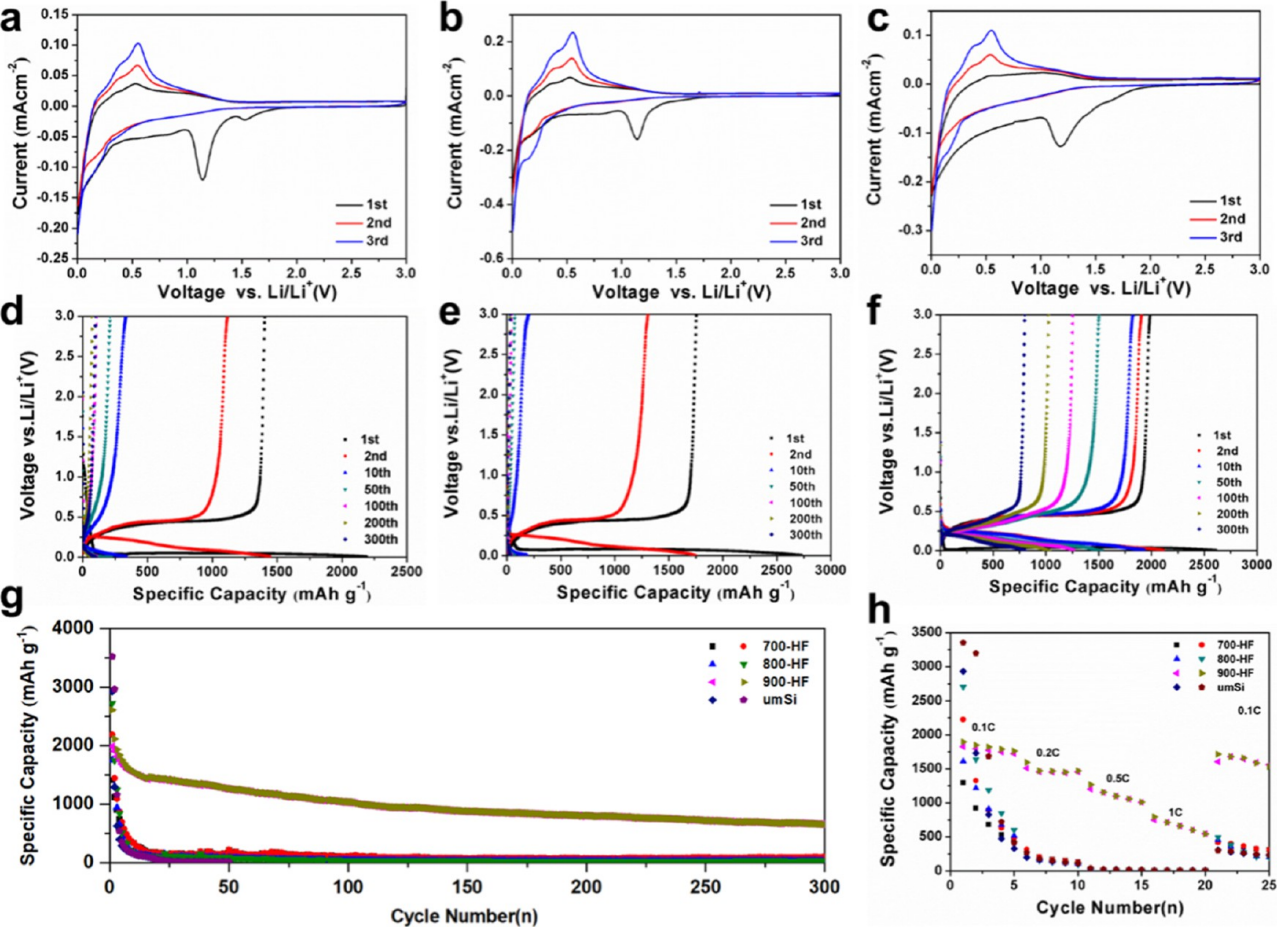
CV curves (a–c), galvanostatic discharge/charge profiles (d–f), cycle performance at 0.2C (g) and rate performance­(h) of 700-HF (a,d), 800-HF (b,e) and 900-HF (c,f).

Figure 6d–f exhibits the discharge/charge profiles of 700-HF, 800-HF, and 900-HF electrodes across 300 cycles (1st, 2nd, 10th, 50th, 100th, 200th, 300th) at 0.2 A g^–1^ within 0.005–3.0 V. The first discharge cycle exhibits an extended voltage plateau near 0.1 V, corresponding to the lithiation-induced alloy formation (Si → Li_*x*_Si). In parallel, the first charging process displays a distinct plateau spanning 0.3–0.5 V, attributed to the delithiation of Li_*x*_Si alloy (Li_*x*_Si → Si). These characteristic voltage plateaus align with the CV results, confirming the typical electrochemical behavior of silicon anodes. Notably, the three porous silicon samples exhibit distinct initial specific capacities and initial Coulombic efficiencies (ICEs): 700-HF demonstrates initial discharge/charge capacities of 2189/1406 mAh g^–1^, corresponding to an ICE of 64.2%, while 800-HF achieves slightly higher discharge/charge capacities of 2728/1754 mAh g^–1^ with a comparable ICE of 64.3%. In contrast, the 900-HF anode stands out by attaining the highest ICE of 76.1%, accompanied by discharge/charge capacities of 2611/1987 mAh g^–1^. This improvement in ICE for the 900-HF sample can be attributed to its well-defined hierarchical macro-/mesoporous architecture. The reduced specific surface area minimizes the extent of electrolyte decomposition and SEI formation during the first lithiation, thereby mitigating irreversible lithium consumption. Furthermore, the continuous 3D silicon network ensures more uniform SEI growth and better electrical connectivity, reducing the risk of localized overlithiation. The underlying interfacial reaction mechanisms are consistent with recent insights into SEI formation and lithium consumption in silicon-based anodes.^54,55^

Figure 6g presents the cycling stability of commercial microsized Si (μm Si)­and the three porous silicon samples (700-HF, 800-HF, 900-HF) over 300 cycles. Commercial microsized silicon exhibited an initial charge capacity of 2930 mAh g^–1^, yet this value dropped to 150 mAh g^–1^ after 10 cycles and to near-zero capacity beyond 30 cycles. In parallel, 700-HF exhibited an initial reversible capacity of 1406 mAh g^–1^, which declined to 332 mAh g^–1^ after 10 cycles and subsequently plateaued below 100 mAh g^–1^ by the 112th cycle, ultimately yielding a capacity retention of 7% after 300 cycles. Meanwhile, 800-HF started at 1754 mAh g^–1^ but dropped to 180 mAh g^–1^ after 10 cycles, retaining only 31 mAh g^–1^ by the 300th cycle. The degradation mechanisms for microsized silicon, 700-HF, and 800-HF stem from their macro-sized dimensions and inadequate porous frameworks, unable to buffer >360% lithiation-induced volume expansion, causing mechanical stress, particle fracture, electrode pulverization, electrical pathway disruption, and SEI rupture/Li^+^ consumption, collectively accelerating capacity decay. Conversely, 900-HF exhibited superior cycling durability, retaining 1031 mAh g^–1^ at 100 cycles and 655 mAh g^–1^ by 300 cycles—a 33% capacity retention relative to its initial value. The significantly improved cyclic stability originates from the synergistic roles of the hierarchical macro-/mesoporous structure: macropores effectively accommodate the volume expansion and thus mitigate the mechanical stress induced by volume change, while mesopores increase the electrolyte-accessible surface area and shorten the lithium-ion diffusion pathways. This dual-pore architecture ensures superior electrode structural integrity and enhanced electrochemical kinetics, collectively contributing to the prolonged cyclic performance.^56,57^

The rate capabilities of commercial microsized silicon and 700-HF, 800-HF, 900-HF across varying current densities are depicted in Figure 6h. Although the commercial microsized Si anode exhibited an initial specific capacity of 1259 mAh g^–1^ at 0.1C (averaged over the first five cycles), its rate capability deteriorated sharply, retaining only 147 mAh g^–1^ at 0.2C, 25 mAh g^–1^ at 0.5C, and 10 mAh g^–1^ at 1C. Upon returning to 0.1C, only 262 mAh g^–1^ was recoverable, underscoring pronounced rate-dependent degradation. Notably, both 700-HF and 800-HF exhibited similar rate behaviors with commercial microsized silicon, with both lagging significantly behind 900-HF. To be detail, at 0.1C, 700-HF and 800-HF achieved average reversible capacities of 774 mAh g^–1^ and 987 mAh g^–1^, respectively. However, their capacities decreased to 182 mAh g^–1^ and 149 mAh g^–1^ at 0.2C, then nearly collapsed at 0.5C. Upon reverting to 0.1C, the capacities rebounded to 355 mAh g^–1^ and 300 mAh g^–1^, recovering merely 46% and 30% of their original values. In sharp contrast, 900-HF delivered remarkable rate adaptability, with average capacities of 1772 mAh g^–1^, 1463 mAh g^–1^, 1103 mAh g^–1^, and 656 mAh g^–1^ at 0.1C, 0.2C, 0.5C, and 1C, respectively. When the current density was resumed to 0.1C, the capacity was restored to 1618 mAh g^–1^, retaining 91.3% of its initial capacity. The results indicate that the macro-/mesopore architecture within the 900-HF sample facilitates electrolyte infiltration and accelerates charge carrier transport, effectively reducing charge polarization and thereby enhancing rate capability. Moreover, its continuous 3D silicon network minimizes grain boundary resistance, further lowering the barriers to charge transport. These combined structural advantages contribute to the significantly improved rate performance.

The morphology change of the porous silicon anodes after 30 cycles was investigated by SEM, as shown in Figure 7. Before cycling, all three electrodes (700-HF, 800-HF, and 900-HF) exhibit uniform and crack-free surfaces (Figure 7a,e,i), indicating comparable initial electrode integrity. After cycling, the 700-HF and 800-HF electrodes display pronounced surface cracks (Figure 7b,f), signifying severe mechanical degradation, while the 900-HF electrode retains a smooth and crack-free surface (Figure 7j), demonstrating its superior structural stability. High-magnification images further reveal that, although the 700-HF and 800-HF samples initially possessed porous architectures before cycling (Figure 7c,g), their porous structures are almost entirely collapsed after cycling and are covered by a thick SEI layer (Figure 7d,h). Remarkably, the 900-HF electrode still maintains a porous framework after cycling (Figure 7k,l), providing direct visual evidence of its effective stress-buffering capability. These postcycling observations unequivocally support the conclusion that the hierarchical macro-/mesoporous architecture uniquely achieved at 900 °C plays a critical role in accommodating volume expansion, mitigating mechanical stress, and preserving structural stability during repeated lithiation.

**Figure 7:**
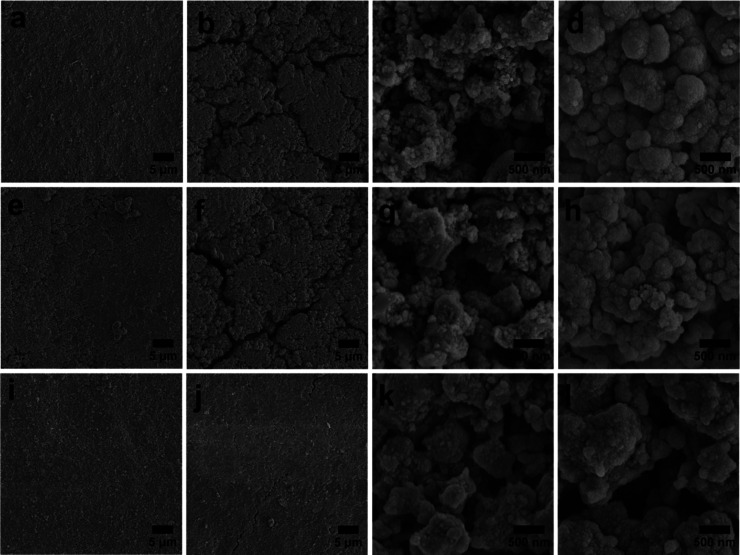
SEM images of 700-HF (a–d), 800-HF (e–h), and 900-HF (i–l) electrodes before (a,c,e,g,i,k) and after (b,d,f,h,j,l) 30 cycles at 0.2C.

The above findings reveal that magnesiothermic reduction temperature plays a pivotal role in determining the electrochemical performance of silicon anodes derived from 730 nm SiO_2_ precursors. Specifically, only when the reduction temperature reaches or exceeds 900 °C can the resultant silicon materials achieve the optimal hierarchical porosity essential for outstanding cyclic stability and rate capability. When the reduction temperature falls below this critical minimum level (e.g., 700–800 °C), the resulting silicon materials demonstrate electrochemical characteristics similar to those of commercial microsized silicon, owing to their structurally inadequate architectures that are unable to effectively accommodate volume changes during cycling. Importantly, the interaction between temperature and particle size uncovers a fundamental limitation in the self-templating process: at the same reduction temperature (e.g., 700 °C), 730 nm SiO_2_ precursors produce fragmented particles with restricted macroporosity, whereas 420 and 130 nm precursors generate robust and well-defined macro/mesoporous networks under identical conditions.^36,37^ This discrepancy highlights that both the precursor particle size and reduction temperature serve as primary factors influencing structural properties.

## 4 Conclusions

In conclusion, this work successfully extends the self-templating magnesiothermic reduction strategy to micron-sized Stöber SiO_2_ by identifying and rationalizing a critical minimum temperature threshold of 900 °C for the formation of robust 3D macro-/mesoporous Si architectures. Systematic characterizations demonstrate that the structural evolution of reduced Si is governed by temperature-activated kinetics designed to overcome the specific diffusion limitations inherent to large, solid SiO_2_ precursors. Reduction below 900 °C results in incomplete conversion and fragmented morphologies due to insufficient Mg vapor penetration and limited Si sintering, thereby leading to poor electrochemical performance in the obtained silicon samples. In contrast, at 900 °C, the enhanced kinetics enable deep Mg penetration and sufficient Si domain fusion, leading to a structurally integral macro-/mesoporous network. This well-defined hierarchical porous architecture effectively accommodates volume expansion and facilitates ion transport, enabling the resulting silicon anode to achieve improved cycling stability (655 mAh g^–1^ after 300 cycles at 0.2C) and rate capability (656 mAh g^–1^ at 1C), which outperforms commercial microsized silicon and samples derived at lower temperatures. Thereby, this study provides a temperature-governed design principle to overcome diffusion limitations and ensure structural integrity when scaling up the self-templating synthesis of hierarchical porous materials.
